# Developmental patterns and characteristics of epicardial cell markers Tbx18 and Wt1 in murine embryonic heart

**DOI:** 10.1186/1423-0127-18-67

**Published:** 2011-08-26

**Authors:** Bin Zeng, Xiao-feng Ren, Feng Cao, Xiao-yang Zhou, Jing Zhang

**Affiliations:** 1 Department of Cardiology, Renmin Hospital of Wuhan University, Wuhan, Hubei, P. R. China; 2 College of Veterinary Medicine, Northeast Agricultural University, Harbin, Hei Longjiang, P. R. China

**Keywords:** Cardiac progenitor, Epicardial cells, Tbx18, Wt1, Development

## Abstract

**Background:**

Although recent studies have highlighted the role of epicardial cells during cardiac development and regeneration, their cardiomyogenic potential is still controversial due to the question of lineage tracing of epicardial cells. The present study therefore aimed to examine the the expression of Tbx18 and Wt1 in embryonic heart and to identify whether Tbx18 and Wt1 themselves expressed in the cardiomyocyte.

**Methods:**

Mouse embryonic hearts were collected at different stages for immunofluorescence costaining with either Tbx18 and the cardiac transcription factor Nkx2.5 or Wilms tumor 1 (Wt1) and Nkx2.5.

**Results:**

Tbx18 and Wt1, but not Nkx2.5, were expressed in the proepicardium and epicardium. Tbx18 was expressed in cells within the heart from E10.5 to at least E14.5; these Tbx18-expressing cells were Nkx2.5 positive, except for a few cells that were Nkx2.5 negative at E14.5. Wt1 was expressed in cells within the heart from E12.5 to at least E14.5, but these Wt1-expressing cells were Nkx2.5 negative.

**Conclusion:**

The data obtained in this study demonstrate that Tbx18 is expressed in the cardiomyocytes from E10.5 to at least E14.5, and Wt1 is expressed within the heart from E12.5 to at least E14.5, but not in the cardiomyocyte. These findings may provide new insights on the role of the epicardial cells in cardiac regeneration.

## Background

During embryogenesis, cells from the proepicardium migrate onto the myocardium to form the epicardium. The proepicardium is a source of undifferentiated progenitor cells that give rise to endothelial cells, fibroblast cells, and the smooth muscle cells that form the coronary vessels during development of the heart [1-3]. The epicardium is the outermost epithelial cell layer overlying the vertebrate heart and was considered historically to be a simple derivative of the proepicardium. However, a series of studies have demonstrated the importance of the epicardium in the development of the heart and in the formation of the coronary vascular system [4,5]. Recently, it was found that the zebrafish epicardium supports cardiac regeneration during the epithelial-to-mesenchymal transition (EMT) and subsequent migration into the myocardium to form new vasculature [6], demonstrating that it could potentially mediate cardiac regeneration after injury in lower vertebrates. Most recently, Tbx18- or Wt1-expressing epicardium was suggested to provide a substantial contribution to myocytes in the ventricular septum (VS) and ventricular walls [7,8], indicating that the epicardium is a potential source for progenitor cells for cardiovascular therapeutics. Because of these recent findings, more and more groups are investigating the regenerative potential of the epicardium because of its cardiomyogenic potential. However, Christoffels et al. show that Tbx18 is expressed in the cardiomyocyte [9], raising a query whether the epicardium may contribute to the cardiomyocyte directly. Although the expression of Tbx18 and Wt1 in heart tissue has been visualized by utilizing genetic knock-in strategies, to the best of our knowledge, localization analyses of the epicardial cell markers Tbx18 and Wt1 in the heart have not been published. In this study, we employed immunofluorescence staining to investigate the expression of Tbx18 and Wt1 in heart using anti-Tbx18 and Wt1 antibodies and to identify whether Tbx18 and Wt1 themselves expressed in the cardiomyocyte.

## Materials and Methods

### Approvement of animal experiments

This study was carried out in strict accordance with the recommendations in the Guide for the Care and Use of Laboratory Animals of the National Institutes of Health. The protocol was approved by the Committee on the Ethics of Animal Experiments of the University of Minnesota (Permit Number: 27-2956). All surgery was performed under sodium pentobarbital anesthesia, and all efforts were made to minimize suffering [10].

### Immunostaining

At least 8 embryos from 5 mice with the same embryonic stages (E9.5, E10.5, E11.5, E12.5, E14.5) were fixed immediately after dissection in 4% paraformaldehyde for 30-60 min. Tissues were embedded in OCT compound (Sigma), then quickly frozen in liquid nitrogen and stored at -80°C. Cryostat sections were cut into 5 μm thin sections. For coimmunostaining, sections were incubated with goat polyclonal anti-Tbx18 (SC-17869, 1:100) and rabbit polyclonal anti-Nkx2.5 (SC-25404, 1:300) or rabbit polyclonal anti-Wt1 (SC-192, 1:300) and goat polyclonal anti-Nkx2.5 (SC-8697, 1:150). The sections were then incubated with the appropriate secondary antibody. The incubation for each primary antibody was performed at 4°C overnight followed by three washes with PBS. Finally, the stained sections were examined using a fluorescence microscope [11,12]. Blocking peptides of Tbx18 (SC-17869 p) and Wt1 (SC-192 p) were used to tested the specificity of Tbx18 and Wt1.

## Results

### Expression of Tbx18 and Wt1 in heart

**Figure 1:**
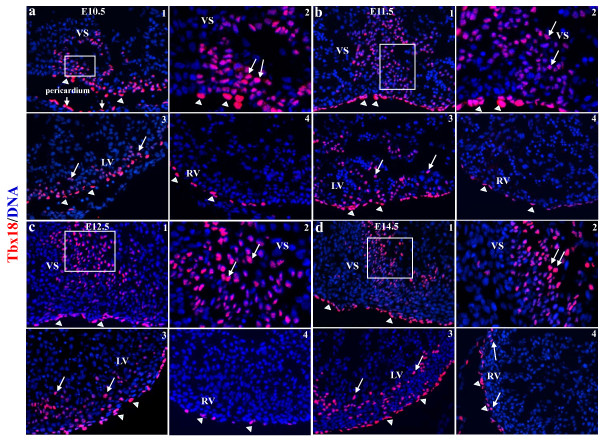
**Expression of epicardial cell marker Tbx18 in murine embryonic heart**. Tbx18 was found not only in the epicardium (arrowheads) but also in the VS and LV from E10.5 to at least E14.5 (arrow), and Tbx18 was also found in the RV at E14.5 (arrow) (a-d). a2, b2, c2, and d2 are higher-magnification views (400×) of a1, b1, c1, and d1 (200×), respectively, and highlight sections from the VS. a3, b3, c3, and d3 are sections from the LV (200×), and a4, b4, c4, and d4 (200×) are sections from the RV. VS, ventricular septum; LA/RA, left/right atrium; LV/RV, left/right ventricle.

**Figure 2:**
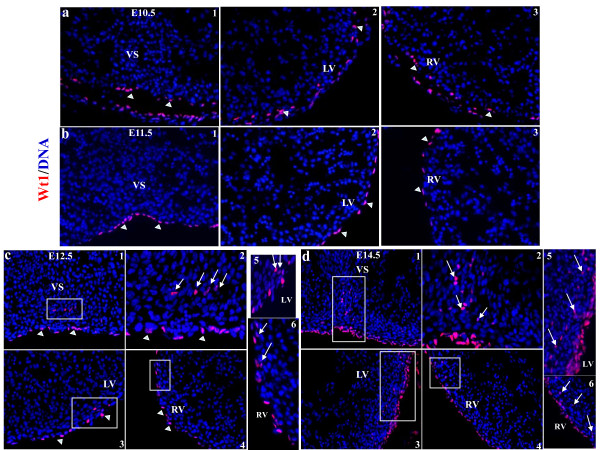
**Expression of epicardial cell marker Wt1 in murine embryonic heart**. Expression of Wt1 was confined to the epicardium (arrowheads) from E10.5 to E11.5 (a, b), but was detected within the heart (arrow) from E12.5 to E14.5 (c, d). a1, b1, c1, and d1 are sections from the VS (200×); c2 and d2 are higher-magnification views (400×) of c1 and d1, respectively. a2, b2, c3, and d3 are sections from the LV (200×). a3, b3, c4, and d4 are sections from the RV (200×); c5 and c6 are higher-magnification views (400×) of the areas within the white squares in c3 and c4, respectively. d5 and d6 are higher-magnification views (400×) of the areas within the white squares in d3 and d4, respectively. VS, ventricular septum; LA/RA, left/right atrium; LV/RV, left/right ventricle. Blue, DAPI staining of nuclei.

Tbx18 and Wt1 were expressed in the proepicardium and epicardium. Tbx18 and Wt1 were expressed early in the proepicardium, and in scattered epicardial cells over the surface of heart at E9.5 (Supplementary Figure 1). Tbx18 and Wt1 were expressed in the epicardial cells covering the heart after E10.5 (Figure 1, 2). Tbx18 began to be expressed in some cells within the VS and left ventricular wall at E10.5, and robust Tbx18 expression was continuously detectable in the VS and left ventricular wall from E11.5 to at least E14.5 (Figure 1a-d). Tbx18-expressing cells were observed in the right ventricular wall at E14.5 (Figure 1d4). Wt1 expression was confined to the epicardium from E9.5 to E11.5 (Supplementary Figure 1 and Figure 2a-b), but Wt1 started to be expressed in some cells within the VS, left ventricular wall, and right ventricular wall at E12.5 (Figure 2c). More Wt1-expressing cells were expressed at E14.5 in the VS, left ventricular wall, and right ventricular wall (Figure 2d). After incubated with an excess of peptide that corresponds to the epitope recognized by Tbx18 and Wt1 antibodies, the staining disappeared (Supplementary Figure 2).

### Do Tbx-18- and Wt-1 express in the cardiomyocyte

**Figure 3:**
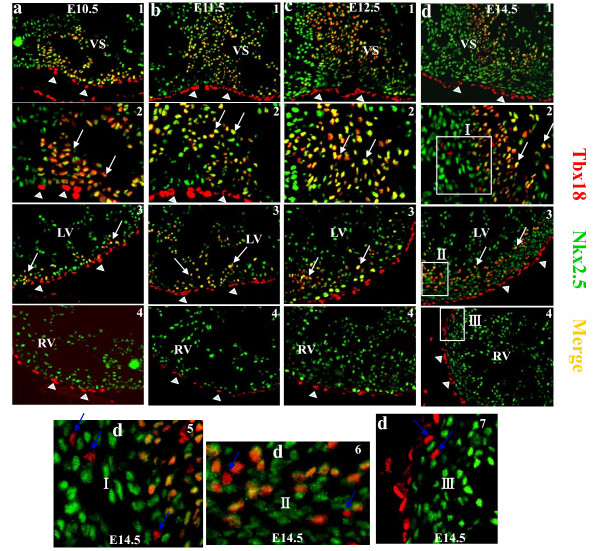
**Characteristics of Tbx18 in murine embryonic heart**. Expression of Tbx18 was expressed in the epicardium (arrowheads) and was also detected in cardiomyocytes in the VS and the LV (white arrow) from E10.5 to E14.5. a1, b1, c1 and d1 are sections from the VS (200×); a2, b2, c2 and d2 are higher-magnification views (400×) of a1, b1, c1 and d1, respectively; a3, b3, c3 and d3 are sections from the LV (200×); a4, b4, c4 and d4 are sections from the RV (200×). d5-7 are higher-magnification views of the white square in d2-4, respectively; they highlight that most of Tbx18-expressing cells are cardiomyocytes, except for a few cells at E14.5 (blue arrow). VS, ventricular septum; LA/RA, left/right atrium; LV/RV, left/right ventricle.

**Figure 4:**
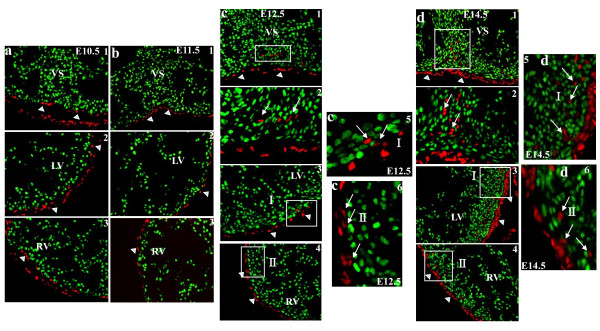
**Characteristics of Wt1 in murine embryonic heart**. Expression of Wt1 was confined to the epicardium (arrowheads) from E10.5 to E11.5 and was also detected within the heart from E12.5 to E14.5 (arrow). a1, b1, c1 and d1 are sections from the VS (200×); c2 and d2 are higher-magnification views (400×) of c1 and d1, respectively. a2, b2, and c3, d3 are sections from the LV (200×). a3, b3, and c4, d4 are sections from the RV (200×). c5,6 and d5,6 are higher-magnification views of the white squares in c3,4 and d3,4 respectively; they highlight that some Wt1-expressing cells are located within the heart but are not cardiomyocytes. Blue, DAPI staining of nuclei.

Coimmunostaining with Tbx18 or Wt1 and the cardiac transcription factor Nkx2.5 showed that Tbx18- and Wt1-expressing epicardial cells were not cardiomyocyte from E9.5 to at least E14.5, because neither were colocalized with Nkx2.5 (Supplementary Figure 2 and Figure 3, 4). Nevertheless, Tbx18-expressing cells in the heart colocalized with Nkx2.5 from E10.5 to at least E14.5 (Figure 3a-d). Interestingly, at E14.5, some Tbx18-expressing cells in the VS and LV did not colocalize with Nkx2.5 (Figure 3d2,3d3, and 3d5,3d6), and Tbx18-expressing cells, as they began to be detected in the right ventricular wall, did not colocalize with Nkx2.5 (Figure 3d4, and 3d7). Wt1-expressing cells began to be detected in some cells within the heart at E12.5 (Figure 4); however, they did not colocalize with Nkx2.5 from E12.5 to at least E14.5 (Figure 4c1-6 and Figure 4d1-6).

## Discussion

The presence of pluripotent cardiac stem cells resident in the myocardium has received considerable attention [13,14]. Recently, epicardial progenitors were identified by expression of Tbx18 and Wt1 due to their cardiomyogenic potential [7,8], and great expectations rasied in the field of cardiac regeneration being relevant to epicardial cells and the transcription factors Wt1 and Tbx18. To obtain better insight into the expression profile and characteristics of the epicardial layer, new mouse epicardial cell marker genes were identified by transcriptomics [15]. Tbx18 and Wt1 have been considered markers of multipotent cardiovascular progenitor cells [16]. Many studies are focusing on epicardial cells, Tbx18 and Wt1. Most of these studies were based on the assumption that the lineage-tracing markers Wt1 and Tbx18 are not expressed in the cardiomyocyte. However, Christoffels et al. showed that cardiomyocytes themselves express Tbx18, challenging the theory that Tbx18-expressing epicardial cells have cardiomyogenic potential [9].

The results of Cai et al.'s study using genetic knock-in strategies showed that Tbx18 was expressed exclusively in the epicardium from E9.5 to E11.5. After E12.5, Tbx18 began to be expressed in some cells within the heart, but it did not colocalize with Nkx2.5. Nevertheless, in our study, we found that Tbx18 expression began in cells within the VS and left ventricular wall at E10.5, and robust Tbx18 expression was continuously detectable in the VS and left ventricular wall from E11.5 to at least E14.5. Furthermore, Tbx18-expressing cells in the heart colocalized with Nkx2.5 from E10.5 to at least E14.5. Our data are consistent with those of Christoffels' group: namely, Tbx18 is expressed in the cardiomyocyte from E10.5 to at least E14.5. Interestingly, we found that, at E14.5, some Tbx18-expressing cells in the VS and LV did not colocalize with Nkx2.5, and Tbx18-expressing cells, as they began to be detected in the right ventricular wall, did not colocalize with Nkx2.5. CFs appear concurrently with ventricular compaction around embryonic day E12.5 and increase in number steadily throughout postnatal day 1 [17]. Those Tbx18-expressing cells, which do not colocalize with Nkx2.5, might be epicardial-derived fibroblast cells. Nkx2.5 is a well-characterized marker of early cardiomyocyte lineage [18]. Though Nkx2.5 expression has been found in progenitors of proepicardium and in coronary vessel smooth muscle cell progenitors [19,20], these studies focused on early stage heart, E9.5. In this study, we found that Tbx18-expressing cells in the heart colocalized with Nkx2.5 from E10.5. The results from Zhou et al.'s study using genetic knock-in showed that Wt1 was confined to the proepicardium and epicardium from E9.5 to E15.5 and not expressed in the cardiomyocyte [8]. However, we found that Wt1 expression was confined to the epicardium from E9.5 to E11.5. Wt1 started to be expressed in some cells within the VS and left ventricular wall at E12.5, but they did not colocalize with Nkx2.5 from E12.5 to at least E14.5.

The failure to detect Tbx18 and Wt1 within the heart may result from the limitations of relying solely on lineage mapping without robust lineage-restricted molecular markers and clear-cut morphological identification criteria [9,21]. Wt1-expressing cells are descended from precursor cells that are positive for Nkx2.5 and Isl1 [8]. Wt1 cardiac conditional knockout mice die between E16.5 and E18.5 due to cardiovascular failure, and *in vitro *Wt1-deficient embryoid bodies lack important markers of endothelial, hematopoietic, and myocardial cells [22]. Wt1 is a crucial gene for the development of epicardial-derived cells; it serves not only to promote the EMT, but also to regulate the origin of the cardiovascular lineage. Though the expression of Wt1 in this study is different from Zhou et al.'s, Wt1 is not expressed in the cardiomyocyte. The differences between Tbx18 and Wt1 expression pattern need further studies to address.

## Conclusions

In conclusion, Tbx18 is expressed in the cardiomyocytes from E10.5 to at least E14.5; Wt1 is expressed within the heart from E12.5 to at least E14.5, but not in the cardiomyocyte. These findings may provide new insights on the role of the epicardial cells in cardiac regeneration.

## Competing interests

The authors declare that they have no competing interests.

## Authors' contributions

BZ designed, carried out the main experiment and drafted the manuscript. XFR helped to improve the manuscript. XYZ and FC helped to finish histological experiments. JZ helped to design the experiment and improve the manuscript. All authors read and approved the final manuscript.
